# Are values related to culture, identity, community cohesion and sense of place the values most vulnerable to climate change?

**DOI:** 10.1371/journal.pone.0210426

**Published:** 2019-01-10

**Authors:** Kristina Blennow, Erik Persson, Johannes Persson

**Affiliations:** 1 Department of Landscape architecture, Planning and Management, Swedish University of Agricultural Sciences, Alnarp, Sweden; 2 Department of Philosophy, Lund University, Lund, Sweden; Universitat de Barcelona, SPAIN

## Abstract

Values related to culture, identity, community cohesion and sense of place have sometimes been downplayed in the climate change discourse. However, they have been suggested to be not only important to citizens but the values most vulnerable to climate change. Here we test four empirical consequences of the suggestion: (i) at least 50% of the locations citizens' consider to be the most important locations in their municipality are chosen because they represent these values, (ii) locations representing these values have a high probability of being damaged by climate change induced sea level rise, (iii) citizens for which these values are particularly strongly held less strongly believe in the local effects of climate change, and (iv) citizens for which these values are particularly strongly held less strongly believe that they have experienced the effects of climate change. The tests were made using survey data collected in 2014 from 326 citizens owning property in Höganäs municipality, Sweden, and included values elicited using a new methodology separating instrumental values from end values, and using the former (which strictly speaking should be seen as estimates of usefulness rather than as aims in themselves) as stepping stones to pinpoint the latter, that represent the true interests of the respondents. The results provide the first evidence that, albeit frequent, values related to culture, identity, community cohesion and sense of place are not the values most vulnerable to climate change. This in turn indicates a need to further investigate the vulnerability of these values to climate change, using a methodology that clearly distinguishes between instrumental and end values.

## Introduction

Assessments of vulnerability to climate change, reflecting exposure, sensitivity and adaptive capacity to climate change [1], typically focus on material and economic aspects of climate change while values related to culture, identity, community cohesion and sense of place are rarely accounted for, e.g. [2–3]. However, these latter kinds of value have been suggested not only important for human well-being but potentially the values most vulnerable to climate change [4], p. 112 in [2].

The citizens themselves might take measures to protect values at risk from climate change, should the society fail to protect them. The preparedness to take measures to adapt has been shown to depend on the decision-making agents' perception of risk, e.g. [5]. Based on a belief-desire model [6–7] driving adaptation, recent studies show that the responses to two questions about the individual, on her or his strength of belief in local effects of climate change and in having experienced the effects of climate change, provide an almost complete explanation to adaptation of forest management to the effects of climate change among private forest owners [8–9]. How strongly an individual believes in the local effects of climate change and in having experienced the effects of climate change are thus important determinants of his or her adaptive measures and hence affects his or her vulnerability to climate change as well.

Also other studies have shown these two factors to be powerful explanatory factors for adaptation to climate change [10–11]. The strength of belief in local effects of climate change provides an explanation to the variation in Swedish municipality planners' implementation of measures for adaptation to climate change as well [12]. In the case of understanding which values are most at risk, past research has found people who particularly value culture, identity, community cohesion and sense of place are less likely to perceive themselves at risk of climate change e.g. [13].

A much tried but often unsuccessful way of making values explicit as well as to make them easier to compare, is to dress them in monetary terms. Adger, O’Brien, and colleagues point out that sometimes "[t]he loss of place and its psychosocial and cultural elements (the loss of a 'world') can arguably never be compensated for with money" [14], p.15 in [4]. The problem can to some extent be dealt with through the use of different kinds of contingent valuation schemes, e.g. [15–18], but when translating non-monetary value into monetary value, some information is inevitably lost. The information lost is typically qualitative information relating to specific types of value, see [19–22] for distinctions between different types of value. In connection with climate change adaptation, qualitative information relating to types of value is sometimes important for actual planning purposes. As has been noted in related fields, see for instance [23–24], it is important to know whether a statement that something has value (end value), means that it is the phenomenon as such that has value or whether it is just another way of saying that this phenomenon is a useful means to promote something else that has value (instrumental value).

There is also a research methodological reason for making the distinction between value as a means to something else and value as an end in itself. Asking respondents about what they value, without making this distinction, leads to a situation where the researchers do not know whether the answer they get represents the degree at which the respondent values the phenomenon itself or whether it represents the degree at which the respondent believes that it is a good way of getting something else that (s)he values. It is therefore important to not just assume that a value answer from a respondent represents what (s)he ultimately values. In order to understand what has value in itself to a respondent, it is thus necessary to ask "why do you value this phenomenon?", and if the respondent answers by pointing at its ability to promote some other phenomenon keep asking until the respondent is no longer prepared to motivate her valuation by pointing at some other phenomenon. In a study that is aimed at understanding values as ultimate motives and not at estimating the more or less educated guesses of the respondents regarding the usefulness of one phenomenon to promote some other phenomenon, it is in fact not meaningful to ask respondents to grade their values until the end value is reached.

What does it mean to say that values related to culture, identity, community cohesion and sense of place are not only important for human well-being but potentially are the values most vulnerable to climate change [4]? A straightforward interpretation would be that those values are important to citizens and especially vulnerable to climate change–for instance since they are not in focus of the authorities’ climate adaptation measures and because the citizens themselves have a low capacity to adapt, that is a low capacity "to prepare for and undertake actions to reduce adverse impacts, moderate harm, or exploit beneficial opportunities" [25], p. 556.

Without making any *a priori* assumptions about the grouping of values with respect to culture, identity, community cohesion and sense of place, here we test the following two hypotheses:

1/ culture, identity, community cohesion and sense of place are highly valued by citizens, and 2/ values related to culture, identity, community cohesion and sense of place are the values most vulnerable to climate change.

More precisely, we tested the following empirical consequences of the two hypotheses:

1 a/ at least 50% of the locations citizens consider to be the most important locations in their municipality are chosen because they represent values related to culture, identity, community cohesion and sense of place,
2 a/ locations representing values related to culture, identity, community cohesion and sense of place have a high probability of being damaged by climate change induced sea level rise, and by assuming that the individual's perception of climate change risk is reflected in his or her strength of belief in local effects of climate change and in having experienced the effects of climate change [8–9], thus affecting his or her vulnerability to climate change, as auxiliary hypotheses:
2 b/ citizens for which culture, identity, community cohesion and sense of place are particularly valuable less strongly believe in the local effects of climate change, and
2 c/ citizens for which culture, identity, community cohesion and sense of place are particularly valuable less strongly believe that they have experienced the effects of climate change.

The empirical consequences of the hypotheses were tested by elicitation of citizens' placebased values and risk perceptions from climate change and comparison with a map of climate change induced sea level rise based on an estimate of the future probability of flooding. The approach to valuation applied is based on developments in value theory [19–22], notably by distinguishing between instrumental and end value. Public participation geographic information system methodology (PPGIS) was used to collect information on the respondents' valuation of the most important location in the municipality, see for instance [26]. The perception of risk from climate change was elicited from the responses to questions asking the citizens about their beliefs about climate change and its effects, cf. [5, 8–9,27]. The results were used to correlate with socio-demographic variables to enable estimation of the prevalence and distribution of value profiles and risk perception in the population, and model risk perception using value profiles and socio-demographic variables as predictors. The usefulness of the results was discussed with respect to democratic planning in the face of climate change.

## Materials and methods

### Study area

**Fig 1:**
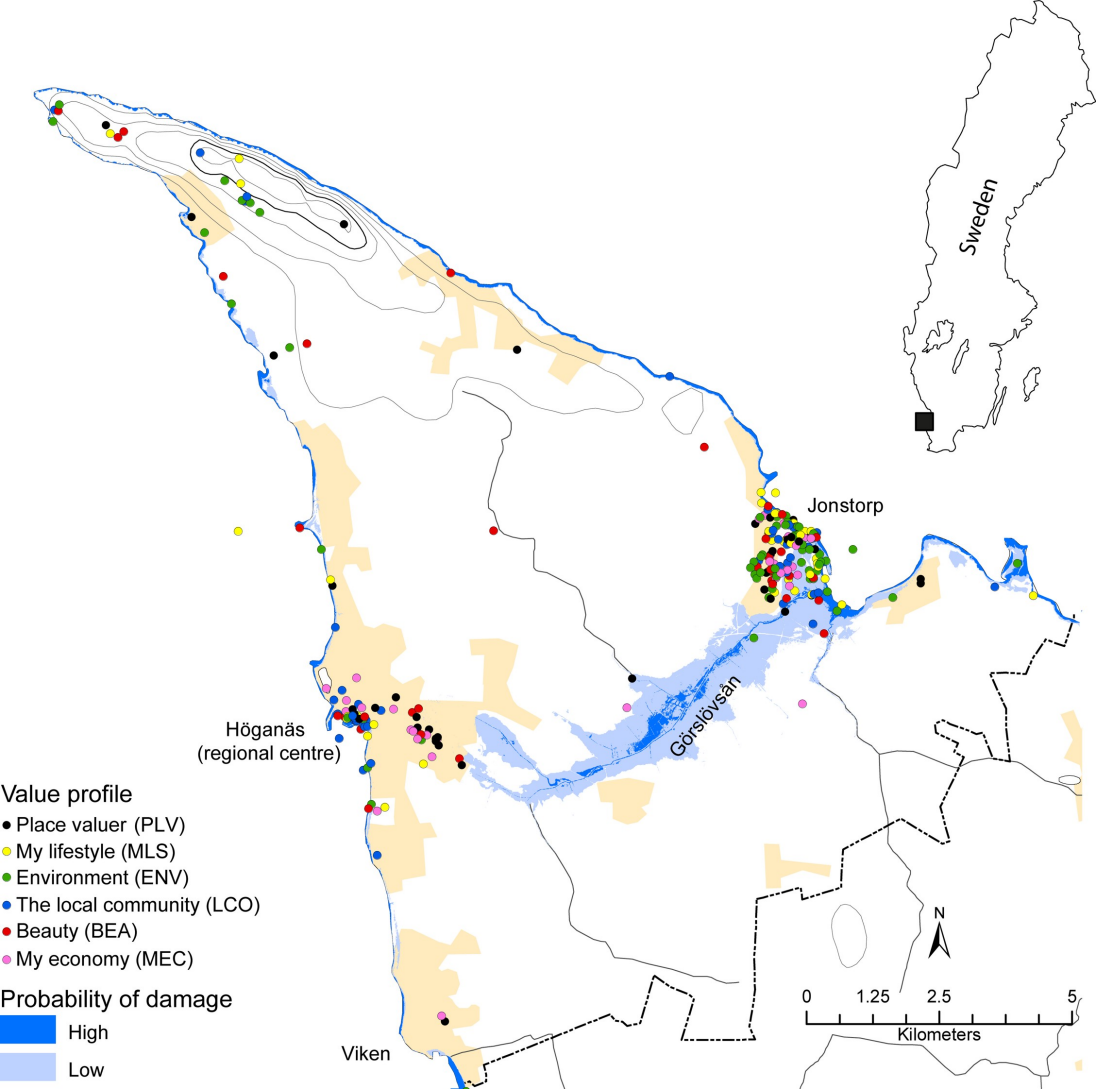
Assessment of the probability of flooding damage and residents' most important location with value profile. The flooding map is based on a model simulation until year 2100 [28]. By "high probability of being damaged" is meant areas flooded permanently by the end of the 21st century and by "low probability of being damaged" is meant areas flooded at a return period of 10 years by the end of the 21st century. The probability of flooding in the remaining areas is estimated to be lower than that. Value profiles were identified based on data before imputation. Inserted map shows the location of the study area in Sweden.

The study was conducted in Höganäs municipality located in southern Sweden (Fig 1). Assuming the local average sea level in year 2100 is +85 cm compared to in 2009, the maximum sea level with a return period of 10 years has been estimated to +237 cm compared to the local average sea level in 2009 [28]. This estimate is based on extreme value statistics applied to sea level observations made in Viken south of the regional centre Höganäs during the period 1976 to 2013 and taking local land rise into account. The rate of post-glacial land rise is lower in southern Sweden than in other parts of Sweden which makes the coast in Höganäs municipality particularly exposed to sea level rise. A 10 year return period corresponds to a probability of temporary flooding in 65% of the years over a 10 year period. The results were distributed across the terrain by use of a digital elevation model of 2 m resolution (Fig 1). The low-lying areas close to the village Jonstorp and along the Görslövsån creek are particularly prone to flooding from sea level rise and the cliff coast in the vicinity of Jonstorp is more susceptible to erosion from sea level rise, see for instance [29], than the coast in the vicinity of Höganäs [30].

### Data collection

All owners of 500 randomly selected properties in each of Höganäs (n = 615) and Jonstorp (n = 695) and all 55 owners of property along the Görslövsån creek (classified as belonging to Jonstorp in the analyses) (Fig 1) were invited by postal mail (with one reminder) to participate in a web-based survey (n = 1365 in total) between 14 October 2014 and 29 December 2014. The invitations were accompanied by a cover letter explaining the objectives of the study and the purpose for which the data collected would be used. Respondents returned the questionnaires voluntarily. A total of 335 persons responded (response rate 25%) of which nine declined participation. Addresses to invitees had been provided by Höganäs municipality. The survey included questions to elicit place-based values, risk perceptions and socio-demographic information and was implemented in the open source web-survey tool LimeSurvey, for instance using its PPGIS functionality [31] (Table 1) (S1 Table).

**Table 1:** Questions assessing respondents' perceptions relating to climate change, and socio-demographic variables and responses.

*Question*	*Response option*	*Lived in Höganäs municipality for up to 23 years (%) (n = 150)*	*Lived in Höganäs municipality for more than 23 years (%) (n = 145)*	*Test statistics*
1. Do you think that the climate is changing because of human induced climate change to the extent that it will affect your environment*? (n = 296)				W = 9254, p = 0.031
	Yes, definitely	50.0	34.5	
	Yes, probably	32.0	42.1	
	I do not know	4.0	7.6	
	Probably not	12.7	13.1	
	Definitely not	1.3	2.1	
2. Did you experience extreme weather or that the climate has changed in a way that you interpret as caused by long-term and global climate change? (n = 296)				W = 12630, p = 0.0090
	Yes, definitely	22.7	20.7	
	Yes, probably	46.0	30.3	
	I do not know	12.7	16.6	
	Probably not	16.0	26.9	
	Definitely not	2.0	5.5	
3. In what way did you experience extreme weather or that the climate has changed in a way that you interpret as caused by long-term and global climate change?** (n = 169)	Responses to an open question among which are found			
	Storm surge	18.4	16.2	χ^2^ = 0.15, p = 0.84
	Coastal erosion	20.4	36.5	χ^2^ = 5.65, p = 0.026
4. When were you born? (n = 297)		Range 1930–1992	Range 1931–1982	t = 6.01, df = 289.1, p<0.00001
		Mean 1960	Mean 1952	
		Median 1962	Median 1951	
5. What is your gender? (n = 297)				χ^2^ = 3.71, p = 0.063
	Woman	54.7	43.4	
	Man	45.3	56.6	
	Other	0.0	0.0	
6. What is your highest qualification? (n = 291)				χ^2^ = 10.93, p = 0.025
	Elementary school	6.0	13.1	
	High school	20.1	30.3	
	Vocational training (post high school)	22.0	18.6	
	University	47.3	33.1	
	Doctoral studies	2.0	2.8	
7. Where in the municipality do you live? (n = 326)				χ^2^ = 0.14, p = 0.72
	Höganäs	38.7	36.6	
	Jonstorp or along Görslövsån creek	61.3	63.4	
8. Do you live (permanently) in the municipality? (n = 295)				χ^2^ = 0.051, p = 0.87
	Permanently	86.0	86.9	
	Spare time	14.0	13.1	

The table is based on raw data before imputation.

* refers to what the respondent perceives as his or her environment.

** question asked only to those responding "Yes, probably" or "Yes, definitely" to question no. 2.

Each recipient was assigned a code to enable targeted reminders to be sent to those who did not reply. To allow researchers to connect a particular answer to a particular respondent, the file containing responses needed to be cross-tabulated, which has not been done at any time. The research adhered to the Personal Data Act (SFS 1998:204). No further approval by the Regional Ethical Review Board (Etikprövningsnämnden) was necessary, which was confirmed in writing by a representative of the Board. The study is based on personal data for which legal restrictions for accessing the data apply. The data is deposited at the Swedish University of Agricultural Sciences, and access to them is regulated by the Public Access to Information and Screcy Act (SFS 2009:400). The research material can be accessed by anyone with a legitimate interest in it. Requests should be addressed to the Swedish University of Agricultural Sciences via registrator@slu.se.

**Fig 2:**
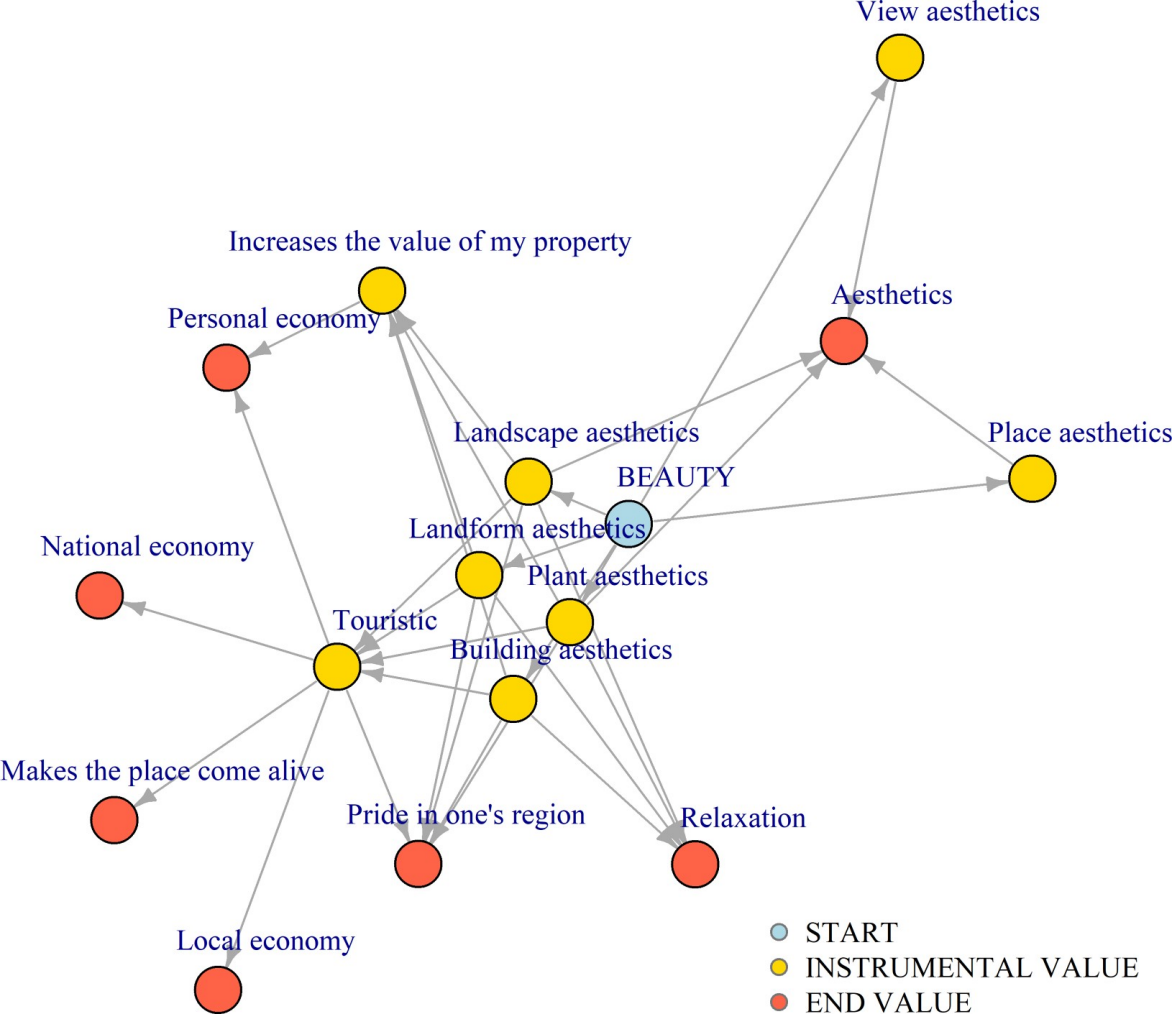
Survey instrument logic for separating instrumental value from end value. The survey instrument uses a chain of questions leading from an initial description of why the chosen location is important to the respondent (START), exemplified in the figure using "Beauty", through a chain of instrumental reasons (INSTRUMENTAL VALUE) leading up to one or more end values (END VALUE). In total the survey instrument uses seven start descriptors, 50 unique instrumental values, and 57 pre-defined end values (S1 Table).

Typically, survey studies are not designed to differentiate between instrumental and end values, which means it is difficult, and sometimes impossible, to know what the answers to value questions in surveys actually tell us. In this study, a special kind of questionnaire was used to come to terms with this problem. Each invitee was asked to mark the location (place or area) within Höganäs municipality most important to him or her using an interactive map. Although an individual's second most valuable location might be almost as important to him or her, by inviting many individuals to provide their most important location a systematic sample of important locations is collected. Subsequent on choosing the most important location the respondent was asked questions to elicit what makes the location important to him or her through answers to a series of questions. Following the choice of location, the first question asked the respondent to select the values that contributed the most to making the location important to him or her. The following categories had been made available to choose from: i/ Good place for bathing, ii/ Beauty, iii/ Exercise, iv/ Nature, v/ Historical/Cultural/Social, vi/ Economic, and vii/ Spiritual. To handle the case where a location represents several end values, multiple categories (up to 3) were allowed. For each category of values chosen, the respondent was asked why that category is important. For instance, a respondent who had chosen the category "Beauty" would be asked to select up to three of the following alternative potential motivations: i/ The place itself is beautiful, ii/ The view is beautiful, iii/ The flora is beautiful, iv/ The landforms are beautiful, v/ The buildings are beautiful, vi/ The landscape of which the location is a part is beautiful. By choosing a certain motivation, new questions were triggered unless the chosen motivation had been predefined as an end value. Hence the questions asked made up one or more chains of questions with each chain leading up to an end value (Fig 2). In total 57 different potential end values were predefined (S1 Table). These had been provided by the authors as expert opinions on which values might be threatened by flooding in the area in question. The respondents were asked to rate the value of each end value chosen on a scale from 1 through 7. The median was used in cases where more than one value was assigned to one end value.

### Data analysis

**Fig 3:**
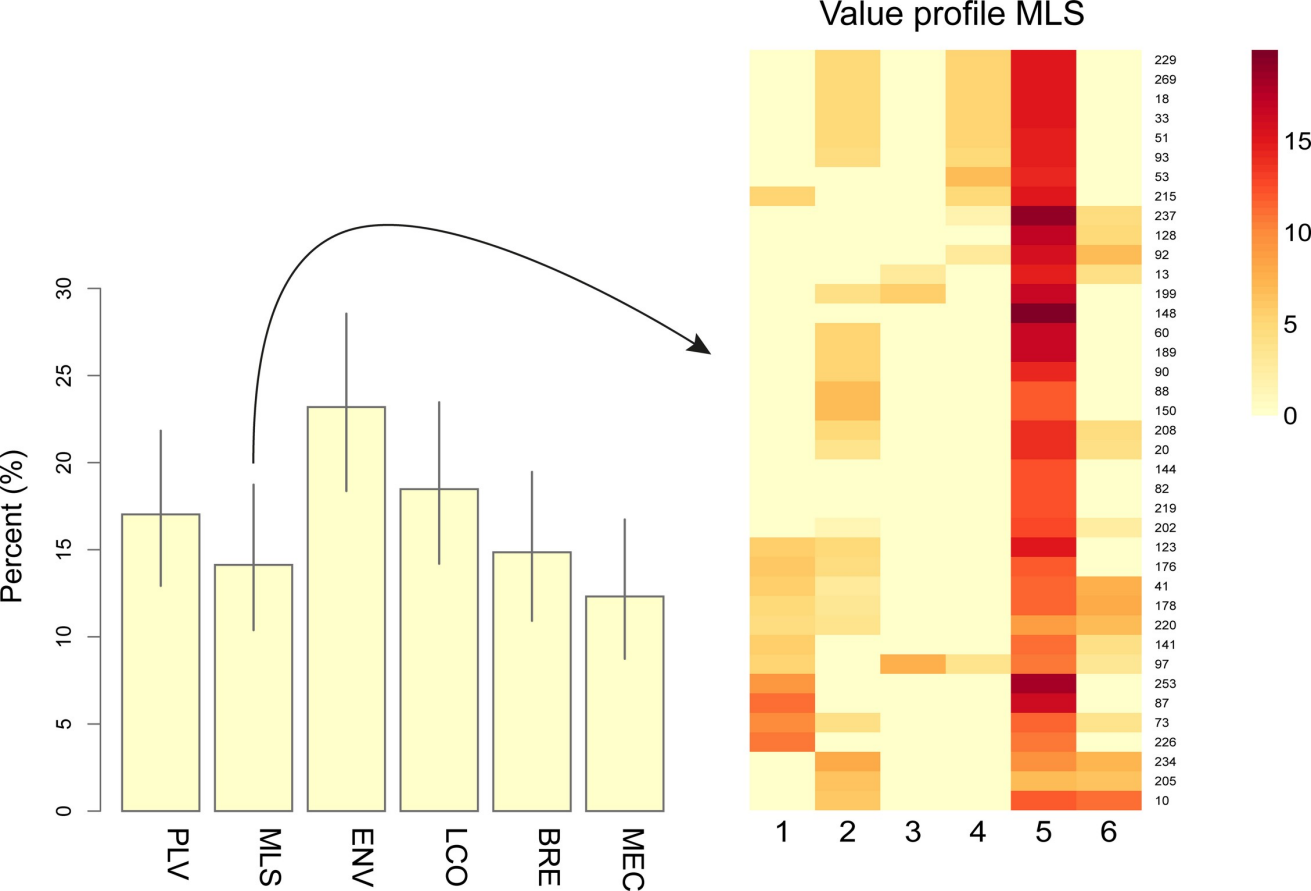
Value profiles in percent of locations chosen. Value profiles for identified groups are based on individual respondents’ preference loadings (S1 Fig) on all value clusters identified (S2 Fig). PLV = "Place valuer", MLS = "My life style", ENV = "Environment", LCO = "The local community", BEA = "Beauty" and MEC = "My economy". Bars represent 95% confidence bands. By way of example, the heatmap shows loadings on value clusters for the 39 respondents with a "My lifestyle" (MLS) value profile. Numbers 1 to 6 refer to value clusters 1 = "Focus on the local community", 2 = "Aesthetics", 3 = "Personal economy", 4 = "The place as such", 5 = "Active and conscious lifestyle choices", and 6 = "Nature and health" (S2 Fig) (S2 Table). Based on data before imputation (n = 276).

Respondents are known to often use scales of measurement that are non-linear and that differ between individuals when rating values [32]. In our study the individuals' valuations were optimally scaled to maximize the sum of the largest eigenvalues [33], the number of which was determined using scree plots. The optimally scaled transformations (>0) were then used to co-cluster the values and respondents/locations using the machine-learning technique of non-negative matrix factorization (NMF), to identify clusters of value items and show how the respondents' valuations were loaded on these, as described in [34] (S1 and S2 Figs) (S1 Table). To enable consideration of different value strengths, the respondents' loadings on the identified clusters of values were used to cluster respondents/locations into groups representing different value profiles using the Affinity Propagation Clustering methodology [35] (Fig 3) (S3 Fig). To handle missing data in modelling, the questions were used as variables to impute five complete data sets (n = 302) using maximum likelihood methodology [36]. Conditional random forest methodology for ordinal data [37] was then applied to all five datasets to estimate the importance in terms of the ranked probability score [38] of value profiles and socio-demographic variables used as predictors of the two belief variables, respectively. This modelling methodology accounts for potential collinearity between predictor variables. The Student’s t-test was used to test for differences between groups described by numeric data, Pearson's χ^2^-test with simulated p value was used to test for differences between groups of categorical data [39], Wilcoxon rank-sum test with continuity correction was used to test for differences between groups described by data at ordinal level, and Kendall's rank correlation tau was used to test for correlations involving data at ordinal level. Average test results were computed over the imputed data sets. All tests were two-tailed and made at α = 0.05. All analyses were conducted using the R Project for Statistical Computing packages v3.3.1 [40], and in particular by applying the libraries Amelia II for multiple imputation [41], "APCluster" for Affinity Propagation Clustering [35], "Aspect" for optimal scaling [42], "party" for conditional random forest modeling [43], "vcd" for visualizing categorical data [44], and "NMF" for nonnegative matrix factorization [45].

## Results

### Type of value profiles

Values related to culture, identity, community cohesion and sense of place contribute to all value profiles identified among the respondents except "My economy" (MEC). They represent more than 50% of the chosen locations in this study (Fig 3) although the value profiles "My lifestyle" (MLS) and "Environment" (ENV) are different from the other value profiles and include aspects of "personal well-being" and "identity" and "a sense of place", respectively (Table 2). "Environment" (ENV) is the single value profile characterizing the largest number of locations chosen (23%) and is statistically significantly more common among respondents living in Jonstorp than in Höganäs (χ^2^ = 9.91, n = 276, p = 0.0019) (see Figs 1 and 3) (S4 Fig). The value profile "The local community" (LCO) is statistically significantly more common among respondents having lived for a period up to the median of 23 years in the municipality than among those having lived in the municipality for a period longer than the median (χ^2^ = 7.48, n = 269, p-value = 0.0068) (S5 Fig).

**Table 2:** Value profile interpretation of respondents’ loadings on value profiles.

*Value profile*	*Interpretation*	*Characterisation in relation to "culture*, *identity*, *community cohesion and sense of place"*, *and other values ("personal well-being"*, *and "economic/material value")*
Place valuer (PLV)	This profile is strongly dominated by the value cluster "The place as such", indicating that the answers that fit this profile are strictly motivated by a concern for the place as such independently of what it is used for.	Sense of place
My lifestyle (MLS)	This profile is strongly dominated by value cluster "Active and conscious lifestyle choices", indicating that the answers that fit this profile are strongly motivated by active and conscious decisions regarding one’s own lifestyle. This profile can therefore not be said to focus on culture or community. Instead, it is clearly focused on the individual but not on traditional material/economic values. Instead it has a strong focus on personal identity and well-being.	Identity/ Personal wellbeing
Environment (ENV)	This profile is strongly dominated by value cluster "Nature and health" which focuses on both the nature in itself and one’s own health. Concern for nature in its own right is often classified under the umbrella term as concern for the environment. The concern for one’s own health in cluster "Nature and health" tends to materialise in a concern for the place constituting a healthy environment.	Sense of place/Personal well-being
The local community (LCO)	This profile is strongly dominated by the value cluster "Focus on the local community" which has to do with local values including the local economy and a wish to liven up the local community.	Community cohesion
Beauty(BEA)	This profile is not strongly dominated by any value cluster but the cluster that is most influential is "Aesthetics", which focuses strongly on aesthetics.	Culture
My economy (MEC)	This profile is strongly dominated by value cluster "Personal economy" which has to do with one’s personal economy. It thus fits well into the traditional focus on economic values.	Economic value

See S3 Fig.

### Locations according to flooding areas

**Fig 4:**
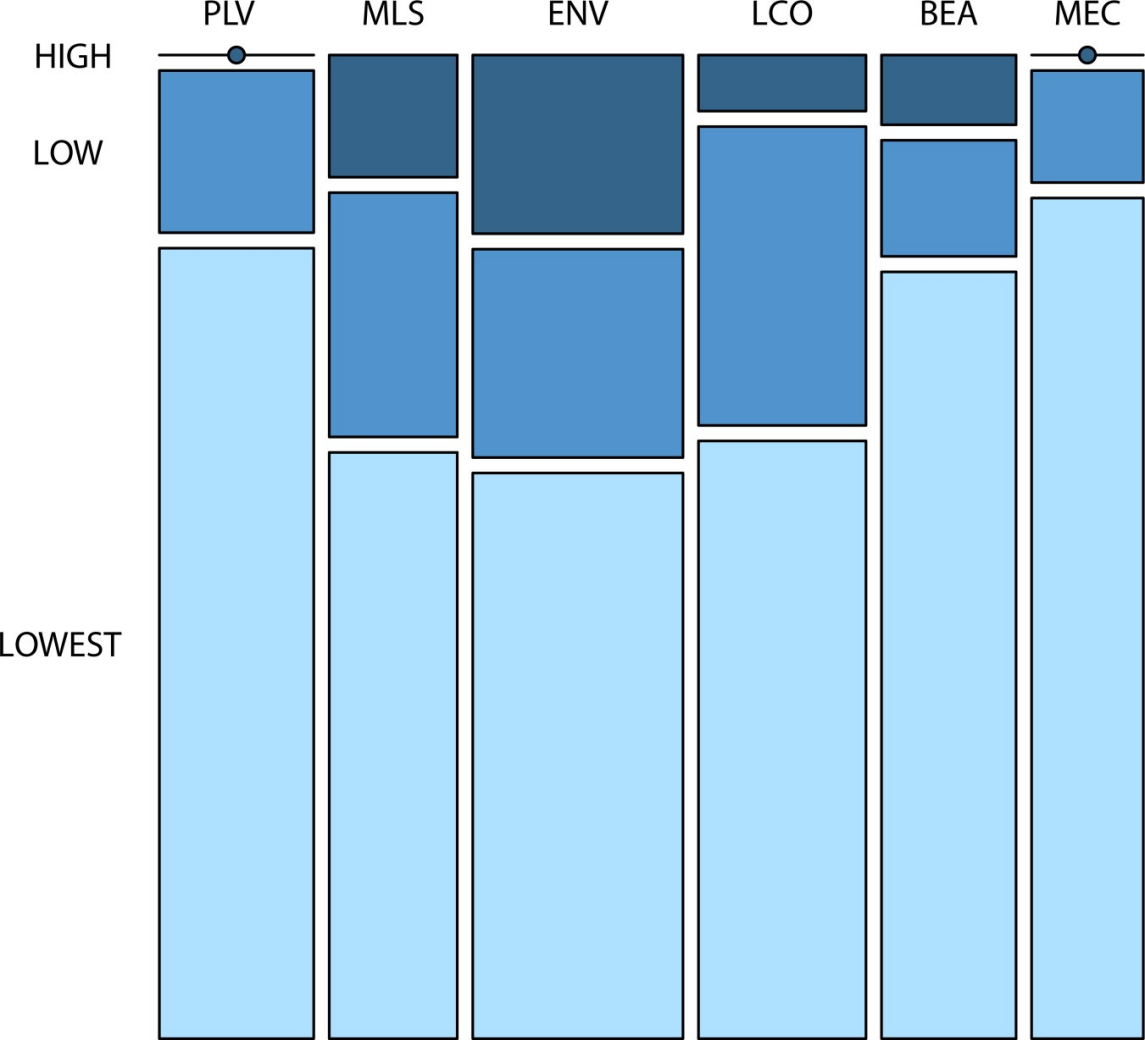
Relationship between value profile of locations selected and the probability of flooding damage. The probability of flooding damage is taken according to the flooding model depicted in Fig 1. The size of the respective compartment is proportional to the number of observations in the respective category. PLV = "Place valuer", MLS = "My lifestyle", ENV = "Environmental", LCO = "The local community", BEA = "Beauty", MEC = "My economy". Based on raw data before imputation.

Twenty-nine percent of the locations chosen are centred in areas prone to flooding by the end of the 21st century according to the flooding model (Fig 1); 21% in areas of low probability and 8% in areas of high probability of flooding (Fig 4). Locations representing the value profile "Environment" (ENV) are significantly more often found in areas with high probability of flooding by the sea by the end of the 21st century than locations represented by other value profiles (W = 7993, n = 276, p = 0.0066) and locations representing the value profiles "Place valuer" (PLV) and "My economy" (MEC) combined are statistically significantly less often found in areas with high probability flooding by the sea by the end of the 21st century than locations represented by other value profiles (W = 6176, n = 276, p = 0.00034). Moreover, locations selected by respondents from Jonstorp are statistically significantly more often found in areas of higher probability of damage by climate change induced sea level rise than locations selected by respondents from Höganäs (see Fig 1) (S6 Fig).

### Risk perception and expected local effects

**Fig 5:**
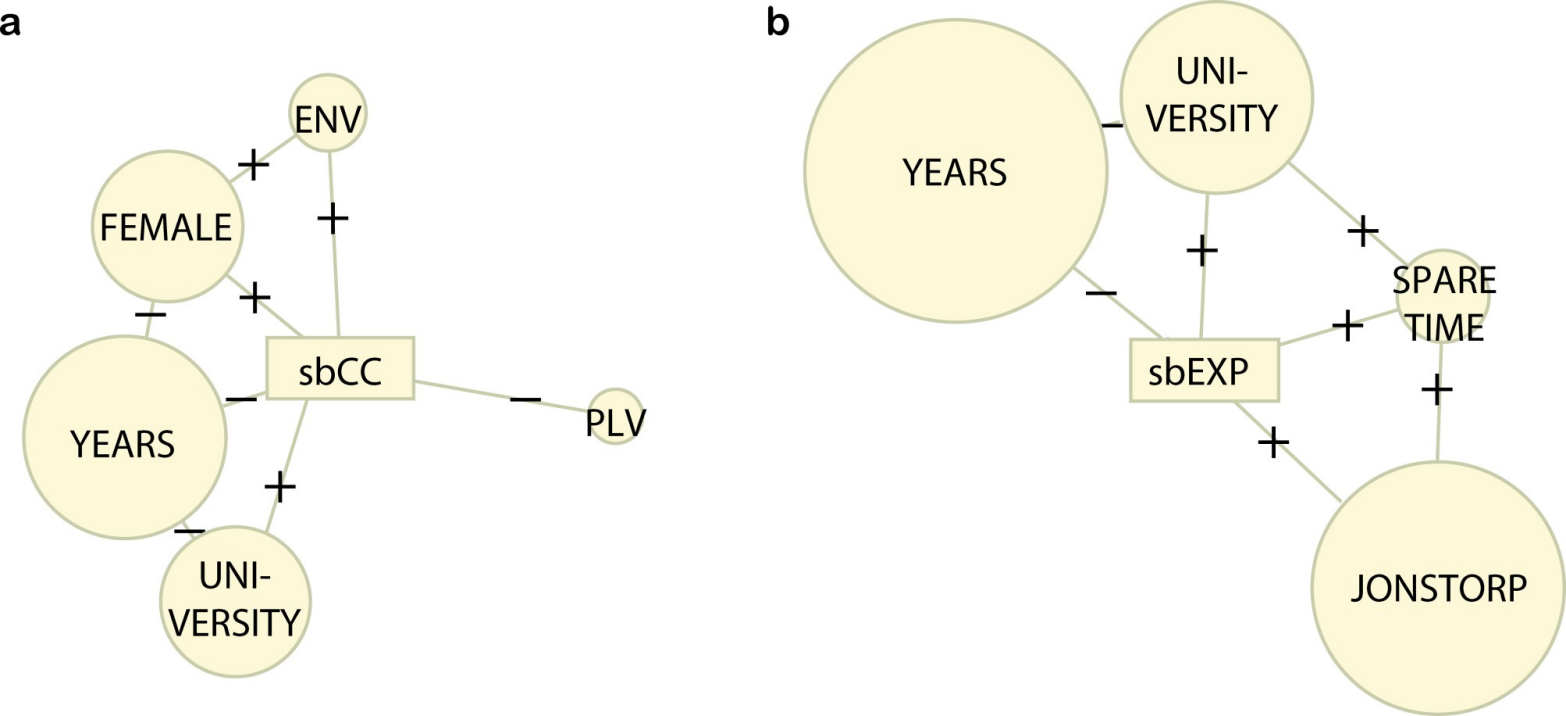
Models of risk perception components. Models of the strength of belief in local effects of climate change. (sbCC) (Question 1 in Table 1) (a) and the strength of belief in having experienced the effects of climate change (sbEXP) (Question 2 in Table 1) (b). The size of the circular nodes represents the variable importance in terms of the ranked probability score of statistically significant predictor variables (S3 Table). The valence of the correlation between variables is represented by + and -, respectively. YEARS represents the number of years lived in Höganäs municipality, UNIVERSITY university education, FEMALE the gender female, PLV and ENV the "Place valuer" and "Environment" value profiles, respectively, JONSTORP resident of the village Jonstorp or along the Görslövsån, and SPARE TIME resident living in the municipality on their spare time only rather than permanently.

Approximately 4 out of 5 respondents answered "Yes, definitely" or "Yes, probably" to the question "Do you think that the climate is changing because of human induced climate change to the extent that it will affect your environment?" (Table 1) (S7 Fig). In a model explaining the response to this question, the number of years the respondent had lived in the municipality is the most important explanatory variable (Fig 5) (S3 Table). The results also show that female respondents and those with university education more strongly believe that they have experienced the effects of climate change than male respondents and those without university education (Fig 5) (S3 Table). Respondents choosing the most important location because of the value profile "Environment" (ENV) statistically significantly more strongly believe in the local effects of climate change than other respondents and respondents choosing the most important location because of the "Place valuer" profile (PLV) statistically significantly less strongly believe in the local effects of climate change than the other respondents (Fig 5) (S3 Table).

### Risk perception and experience of local effects

Approximately 3 out of 5 respondents answered "Yes, definitely" or "Yes, probably" to the question "Did you experience extreme weather or that the climate has changed in a way that you interpret as caused by long-term and global climate change?" (Table 1) (S8 Fig). In a model explaining the response to this question, the number of years the respondent had lived in the municipality is the most important explanatory variable with those having lived in the municipality for up to the median 23 years more strongly believing that they have experienced the effects of climate change than those having lived in the municipality for more than 23 years (Fig 5) (S3 Table). The results also show that respondents living in Jonstorp, those with university education, and those living in the municipality in their spare time only, more strongly believe that they have experienced the effects of climate change than respondents living in Höganäs, those having no university education and those living in the municipality permanently do (Fig 5) (S3 Table). No statistically significant association was found between the strength of belief in having experienced the effects of human induced climate change and value profile.

Among respondents who answered "Yes, definitely" or "Yes, probably" to the question "Did you experience extreme weather or that the climate has changed in a way that you interpret as caused by long-term and global climate change?", 17% reported having experienced climate change induced storm surge and 27% reported having experienced climate change induced coastal erosion (S9 Fig). Respondents having lived in the municipality for a period longer than 23 years (median) statistically significantly more often reported that they have experienced climate change induced coastal erosion than those having lived in the municipality for a period up to the median did (S9 Fig). The results also show that respondents living in Jonstorp statistically significantly more often reported that they have experienced climate change induced coastal erosion than those living in Höganäs did (S10 Fig).

## Discussion

Values related to culture, identity, community cohesion and sense of place have sometimes been downplayed in climate change discourse. However, they have also been suggested not only important for human well-being but potentially the values most vulnerable to climate change [4]. We tested four empirical consequences (1a, 2a, 2b, and 2c) of the two hypotheses 1/ culture, identity, community cohesion and sense of place are highly valued by citizens and 2/ values related to culture, identity, community cohesion and sense of place are the values most vulnerable to climate change. This was done by asking citizens living in Höganäs municipality, Sweden, questions relating to place-based values and perceptions in relation to climate change induced sea level rise using a new survey instrument and comparing the results to a model estimate of the future probability of flooding [28] (Fig 1). The survey instrument uses a new questionnaire structure that for the first time makes it possible to separate instrumental values from end values (Fig 2). The importance of separating instrumental values from end values has been advocated e.g. by The Intergovernmental Science-Policy Platform on Biodiversity and Ecosystem Services [24].

Most of the important locations chosen by the respondents in this study can be characterised as representing values related to culture, identity, community cohesion and sense of place (Fig 3) (Table 2) and hence the empirical consequence 1 a/ saying that at least 50% of the locations citizens consider to be the most important locations in their municipality are chosen because they represent values related to culture, identity, community cohesion and sense of place was corroborated. Indeed, only the value profile "My economy" (MEC) representing 12% of the respondents is unrelated to these values (Fig 3) (Table 2). The value profile "The local community" (LCO) is more common among those who have lived in the municipality for a period up to the median (23 years) than among those who have lived in the municipality for a period longer than the median 23 years (S5 Fig). This is in agreement with the findings of a study on coastal Australian communities even though in that study the valuation scheme used did not separate instrumental values from end values [44].

Value elicitation is a complicated task, e.g. [24]. One factor that tends to complicate comparisons is that different authors use different value classifications. The classification used by Adger, O’Brien and colleagues [2–4] is based on categories (culture, identity, community cohesion and sense of place) that did not exist in any of the classifications developed by Rokeach, Schwartz and others, see [16] for a listing of the latter, that have served as a kind of standard for empirical value studies. The negative effects on comparability have to be weighed against the new knowledge that can be gained through the development of new classifications, however, and different classifications are sometimes necessary for different questions. The standard classifications have turned out to have a great explanatory power, but do not help us in the task at hand. Adger and O’Brien’s classification [2–4] is clearly useful for its purpose even though it makes comparisons with older studies more difficult.

One aspect of the classification by Adger and O'Brien [2–4] that is potentially problematic, however, is its inability to distinguish between instrumental value and end value, or means and ends see e.g. [24], which we deem very important for decisions about climate change adaptation. We have therefore chosen to make use of this distinction in our study while still using Adger, O’Brien’s classification scheme [2–4] for classifying the resulting value profiles. This means our classifications are comparable though the distinction between means and ends in addition makes it possible for us to know for instance if a chosen beach location is important because it provides opportunity to go swimming or if the location has value in its own right. If the location has instrumental value for swimming and not as end in itself, then perhaps new beaches can be created as the sea level rises that will provide opportunity for swimming. On the other hand, if the beach location is important in its own right, then that end value will be lost when the location is flooded by the sea. Moreover, by repeatedly asking the respondents why their answer is important until an end is reached, a very different answer can result compared to after the first iteration. For example, some respondents in our study reported to have chosen the most important location because of its beauty in the first iteration and not until after several iterations it was revealed that the end value is related to the respondents’ personal economy and not the beauty as such, perhaps from earning a living on tourists visiting the beautiful location (Fig 2). This makes it difficult to compare the results of this study with value elicitation studies not designed to account for the difference between instrumental values and end values, e.g. [46–48].

Locations representing the value profile "My economy" (MEC) and the value profile "Place valuer" (PLV) are only observed outside of the areas of high probability of climate change induced damage from sea level rise according to the flooding model we have used (dark blue areas in Fig 1) while locations representing "Environment" (ENV) is the value profile that is the most often found in areas of high probability of damage from sea level rise (Fig 1 and Fig 4). Although the value profile "My economy" (MEC) is the value profile most strongly in contrast to values related to culture, identity, community cohesion and sense of place, the "Place valuer" (PLV) value profile is clearly related to these values and the value profile "Environment" (ENV) is a mix of material values and values relating to "a sense of place" (Table 2). This means that although the value profiles representing values related to culture, identity, community cohesion and sense of place are frequent they are not more often found in locations of high probability of flooding from sea level rise. Hence, we find that the empirical consequence 2 a/ saying that locations representing values related to culture, identity, community cohesion and sense of place have a high probability of being damaged by climate change induced sea level rise could not be corroborated. It appears reasonable, however, to expect the result to depend on the damage trigger. Indeed, Raymond and Brown [47] found that the spatial overlap of elicited place-based citizen values and modelled high probability of damage from climate change differs depending on the damage trigger; bush-fire, land erosion, wave action, or sea-level rise.

To the extent that 2a is rejected and we accept the auxiliary assumptions it builds on, hypothesis 2 has to be abandoned as an explanation of the phenomenon we study. It is however still possible that locations representing values related to culture, identity, community cohesion and sense of place are more vulnerable than those locations related to other values if citizens who value locations for the former reasons have lower preparedness to take action to protect these places.

Positive responses to the questions "Do you think that the climate is changing because of human induced climate change to the extent that it will affect your environment?" (approximately 4 out of 5 respondents) (Table 1) (S7 Fig) and "Did you experience extreme weather or that the climate has changed in a way that you interpret as caused by long-term and global climate change?" (approximately 3 out of 5 respondents) (Table 1) (S8 Fig) indicate a perception of climate change risk and thereby a capacity to take measures to adapt to climate change [8–11,27]. Few studies report on climate change beliefs of citizens in coastal communities, e.g. [49–51]. In a recent study conducted in the UK, an approximately similar fraction of respondents as in the present study report that they believe in the local effects of climate change although in that study the climate change effect was specified to be sea level rise [52]. The risk perception appears, however, largely unrelated to the value profile (Fig 5) although statistically significant correlations between respondents representing these values were found for those who least strongly (value profile PLV) as well as among those who most strongly (value profile ENV) believe in the local effects of climate change (S3 Table). Hence, we find that 2 b/ saying that citizens for which culture, identity, community cohesion and sense of place are particularly valuable less strongly believe in the local effects of climate change could not be corroborated (Fig 5). Because the experience of climate change effects was found to be unrelated to values (Fig 5) (S3 Table) 2 c/ saying that citizens for which culture, identity, community cohesion and sense of place are particularly valuable less strongly believe that they have experienced the effects of climate change, respectively, could not be corroborated. In a previous study, no clear correlation between climate change risk perception and value profiles was found [53]. Both results suggest that cultural cognition thinking, e.g. [54], have limited explanatory potential.

By asking people to choose the most important location in their municipality and to motivate the choice provided new and useful information for spatial planning, including planning of climate change adaptation and communication. For instance, basing climate change communications on the protection against local risks has been suggested to improve the effectiveness of communications [55]. Furthermore, whether a beach location is important in its own right or as a place to go swimming makes a difference when prioritising and choosing adaptation measures. That values most often are not critical drivers of adaptation decisions makes value elicitation all the more important for climate change adaptation planning that aims at promoting and protecting the citizens´ values. Correlation with socio-demographic variables generated additional information even if a higher response rate would have been desirable for assessing the distribution of important locations and prevalence of value profiles in the population in a truly representative way.

The most important locations chosen by respondents living in Jonstorp are more often threatened by sea level rise than the locations chosen by respondents living in Höganäs according to the flooding model we have used (Fig 1) (S6 Fig). Many of the most important locations chosen by the respondents are found in or in the vicinity of Höganäs and Jonstorp urbanised areas with Jonstorp to a larger extent than Höganäs being located in low-lying and flooding prone areas (Fig 1). Also statistically significantly more respondents from Jonstorp chose the most important locations because of the environmental (ENV) value profile (S4 Fig) which was often found in areas of high probability of flooding damage (Fig 4). However, the vulnerability of the values by respondents living in Jonstorp is potentially reduced because of their higher preparedness to take measures to adapt compared to respondents from Höganäs (Fig 5) (S3 Table). This is because respondents from Jonstorp more often strongly believe that they have experienced the effects of climate change. In particular, respondents from Jonstorp more often report having experienced climate change induced coastal erosion (S10 Fig). This belief is in agreement with the coastline in the vicinity of Jonstorp being more susceptible to undermining and coastal erosion because of ongoing sea level rise than the coastline in the vicinity of Höganäs [28]. Finally, it is noteworthy that citizens who have lived longer in the municipality and presumably have more observational data to rely on significantly less strongly believe in the local effects of climate change and have a weaker strength of belief in having experienced the effects of climate change than the other respondents (Fig 5) (S3 Table).

## Conclusions

A new methodology separating instrumental values from end values has been presented, thus enabling elicitation of the true interests of the respondents. Using the new methodology, we found that values related to culture, identity, community cohesion and sense of place are important to citizens in Höganäs municipality which is in agreement with the straightforward interpretation of the possibility suggested by Adger, O’Brien, and colleagues [2–4]. However, locations representing these values are not more often found in areas of high probability of flooding from climate change induced sea level rise according to the flooding model we have used. Also with respect to the preparedness to reduce vulnerability, we find that respondents with value profiles reflecting values related to culture, identity, community cohesion and sense of place are not less prepared to take measures to adapt than other respondents. We conclude that albeit frequent, values related to culture, identity, community cohesion and sense of place are not the values most vulnerable to climate change. This in turn indicates a need to further investigate the vulnerability of these values to climate change, using a methodology that clearly distinguishes between instrumental and end values.

## Supporting information

S1 Fig Loadings on value clusters estimated for valuations. Valuations by respondents (rows) across 200 runs and sorted according to the maximum value per respondent. Numbers 1 to 6 refer to value clusters 1 = "Focus on the local community", 2 = "Aesthetics", 3 = "Personal economy", 4 = "The place as such", 5 = "Active and conscious lifestyle choices", and 6 = "Nature and health" (S2 Table).
(TIF)

S2 Fig Clusters identified for optimally scaled valuations. 54 end values were selected by the respondents among 57 predefined end values (S3 Table) across 200 runs. Numbers 1 to 6 refer to value clusters 1 = "Focus on the local community", 2 = "Aesthetics", 3 = "Personal economy", 4 = "The place as such", 5 = "Active and conscious lifestyle choices", and 6 = "Nature and health" (see S2 Table).
(TIF)

S3 Fig Value profiles for identified groups. Groups are identified based on individual respondents' preference loadings (S1 Fig) on all value clusters identified (S2 Fig). Numbers 1 to 6 refer to value clusters 1 = "Focus on the local community", 2 = "Aesthetics", 3 = "Personal economy", 4 = "The place as such", 5 = "Active and conscious lifestyle choices", and 6 = "Nature and health" (S2 Table). Elaborate interpretations of the value profiles are provided in Table 2.
(TIF)

S4 Fig Value profile by location. The size of the respective compartment is proportional to the number of observations in the respective category. PLV = "Place valuer", MLS = "My life style", ENV = "Environmental", LCO = "The local community", BEA = "Beauty", MEC = "My economy". The graph is based on raw data before imputation (χ = 12.96, n = 276, p = 0.023).
(TIF)

S5 Fig Relationship between value profile of locations chosen and for how long time the respondent has lived in Höganäs municipality. The size of the respective compartment is proportional to the number of observations in the respective category. PLV = "Place valuer", MLS = "My life style", ENV = "Environmental", LCO = "The local community", BEA = "Beauty", MEC = "My economy". The graph is based on raw data before imputation (χ^2^ = 11.90, n = 269, p = 0.035).
(TIF)

S6 Fig Relationship between where the respondents live in the municipality and probability of flooding because of climate change induced sea level rise. The graph is based on raw data before imputation (W = 10340, n = 324, p = 0.00067).
(TIF)

S7 Fig Relationship between value profile of locations selected and responses to the question "Do you think that the climate is changing because of human induced climate change to the extent that it will affect your environment?" 1 refers to "Definitely not""; 2 "Probably not"; 3 "I do not know"; 4 "Yes, probably" and 5 "Yes, definitely". The size of the respective compartment is proportional to the number of observations in the respective category. PLV = "Place valuer", MLS = "My life style", ENV = "Environmental", LCO = "The local community", BEA = "Beauty", MEC = "My economy".
(TIF)

S8 Fig Relationship between the value profile of locations selected and responses to the question "Did you experience extreme weather or that the climate has changed in a way that you interpret as caused by long-term and global climate change?" 1 refers to "Definitely not""; 2 "Probably not"; 3 "I do not know"; 4 "Yes, probably" and 5 "Yes, definitely". The size of the respective compartment is proportional to the number of observations in the respective category. PLV = "Place valuer", MLS = "My life style", ENV = "Environmental", LCO = "The local community", BEA = "Beauty", MEC = "My economy".
(TIF)

S9 Fig **Relationship between for how long the respondent has lived in the municipality and reports of having experienced storm surge (a) and coastal erosion (b) because of climate change, respectively.** Only responses from those respondents who answered "Yes, definitely" or "Yes, probably" to the question "Did you experience extreme weather or that the climate has changed in a way that you interpret as caused by long-term and global climate change?" were used (Table 1). The is no statistically significant difference between respondents having lived in the municipality for more than 23 years (median) or up to 23 years with respect to reporting to have experienced climate change induced storm surge (χ^2^ = 3.51, n = 179, p = 0.085). Respondents having lived in the municipality for more than 23 years (median) statistically significantly more often reported that they have experienced climate change induced coastal erosion than those having lived in the municipality for a period up to the median did (χ^2^ = 17.81, n = 177, p = 0.00002). Bars represent observations. Based on raw data before imputation.
(TIF)

S10 Fig Relationship between where the respondents live in the municipality and reports of having experienced coastal erosion because of climate change. Only responses from those who answered "Yes, definitely" or "Yes, probably" to the question "Did you experience extreme weather or that the climate has changed in a way that you interpret as caused by longterm and global climate change?" (Table 1). Respondents living in Jonstorp statistically significantly more often reported that they have experienced climate change induced coastal erosion than those living in Höganäs did (χ^2^ = 17.81, n = 179, p = 0.00007). Based on raw data before imputation.
(TIF)

S1 Table Predefined end values and classification. Classification is made in relation to the value categories 'culture', 'identity', 'community cohesion' and 'sense of place' in [3], the range observed (maximum 1–7) and median score of values assigned by n respondents (total n = 276).
(PDF)

S2 Table Value cluster interpretation. (PDF)

S3 Table Statistically significant relationships among variables in the models of risk perception components. The components are strength of belief in local effects of climate change (Question 1 in Table 1) and the strength of belief in having experienced the effects of climate change (Question 2 in Table 1).
(PDF)
