# Supplementary figures and images for: Are values related to culture, identity, community cohesion and sense of place the values most vulnerable to climate change?
Source: PLoS One. 2019 Jan 10;14(1):e0210426. doi: 10.1371/journal.pone.0210426 (PMC6328185; doi:10.1371/journal.pone.0210426)

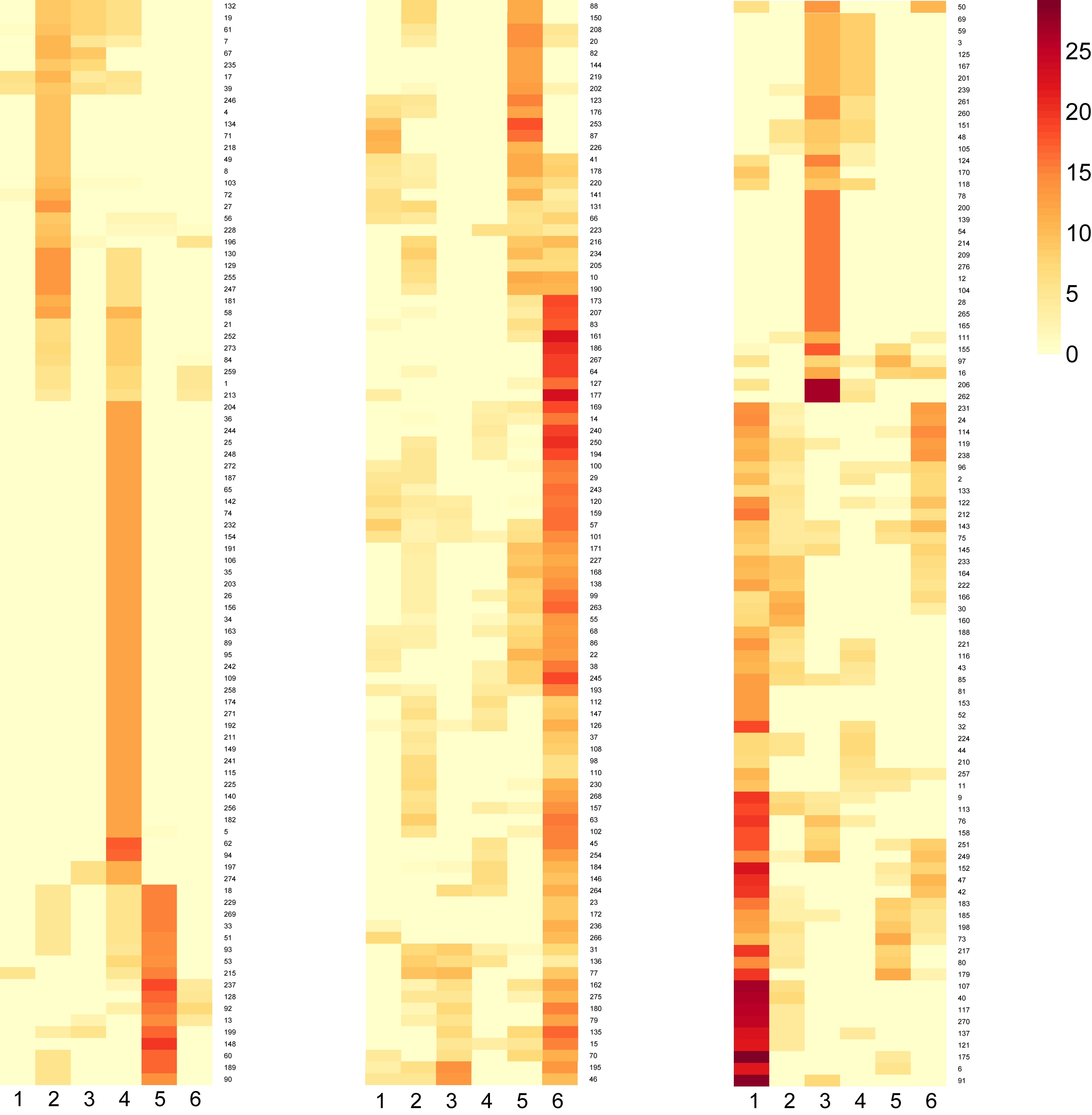

Supplement: S1 Fig — Valuations by respondents (rows) across 200 runs and sorted according to the maximum value per respondent. Numbers 1 to 6 refer to value clusters 1 = "Focus on the local community", 2 = "Aesthetics", 3 = "Personal economy", 4 = "The place as such", 5 = "Active and conscious lifestyle choices", and 6 = "Nature and health" (S2 Table). (TIF) [file pone.0210426.s001.tif]

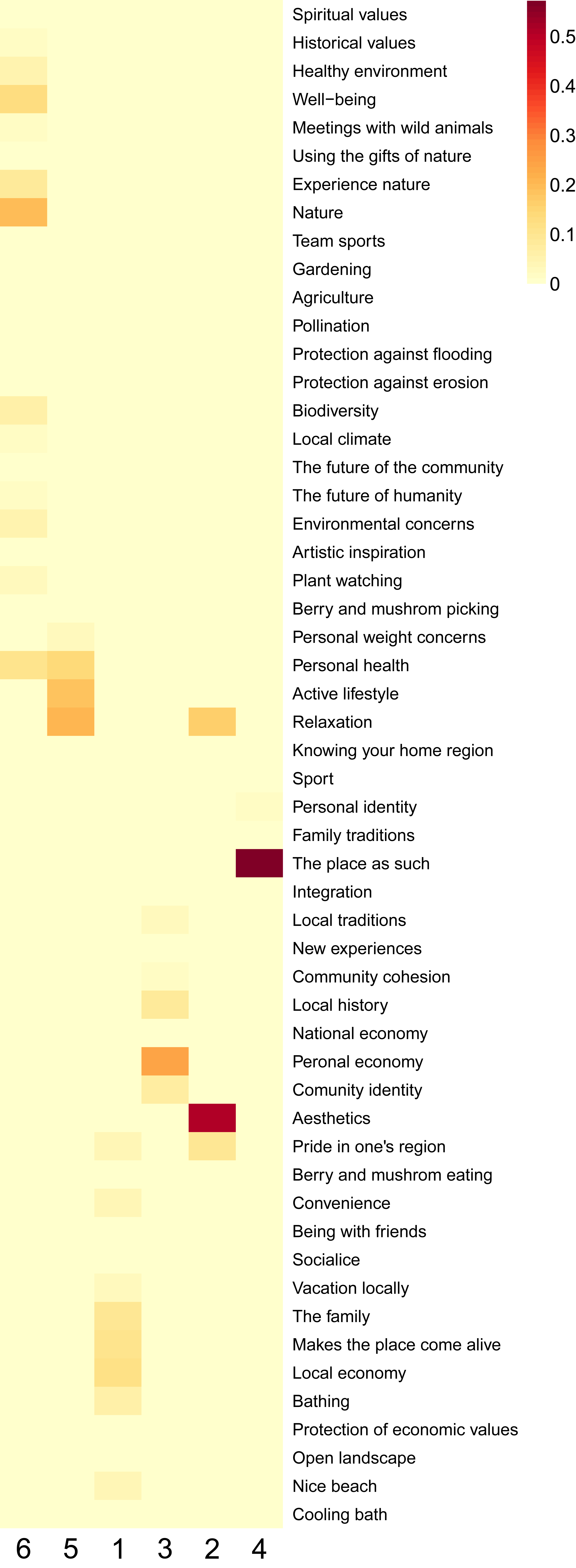

Supplement: S2 Fig — 54 end values were selected by the respondents among 57 predefined end values (S3 Table) across 200 runs. Numbers 1 to 6 refer to value clusters 1 = "Focus on the local community", 2 = "Aesthetics", 3 = "Personal economy", 4 = "The place as such", 5 = "Active and conscious lifestyle choices", and 6 = "Nature and health" (see S2 Table). (TIF) [file pone.0210426.s002.tif]

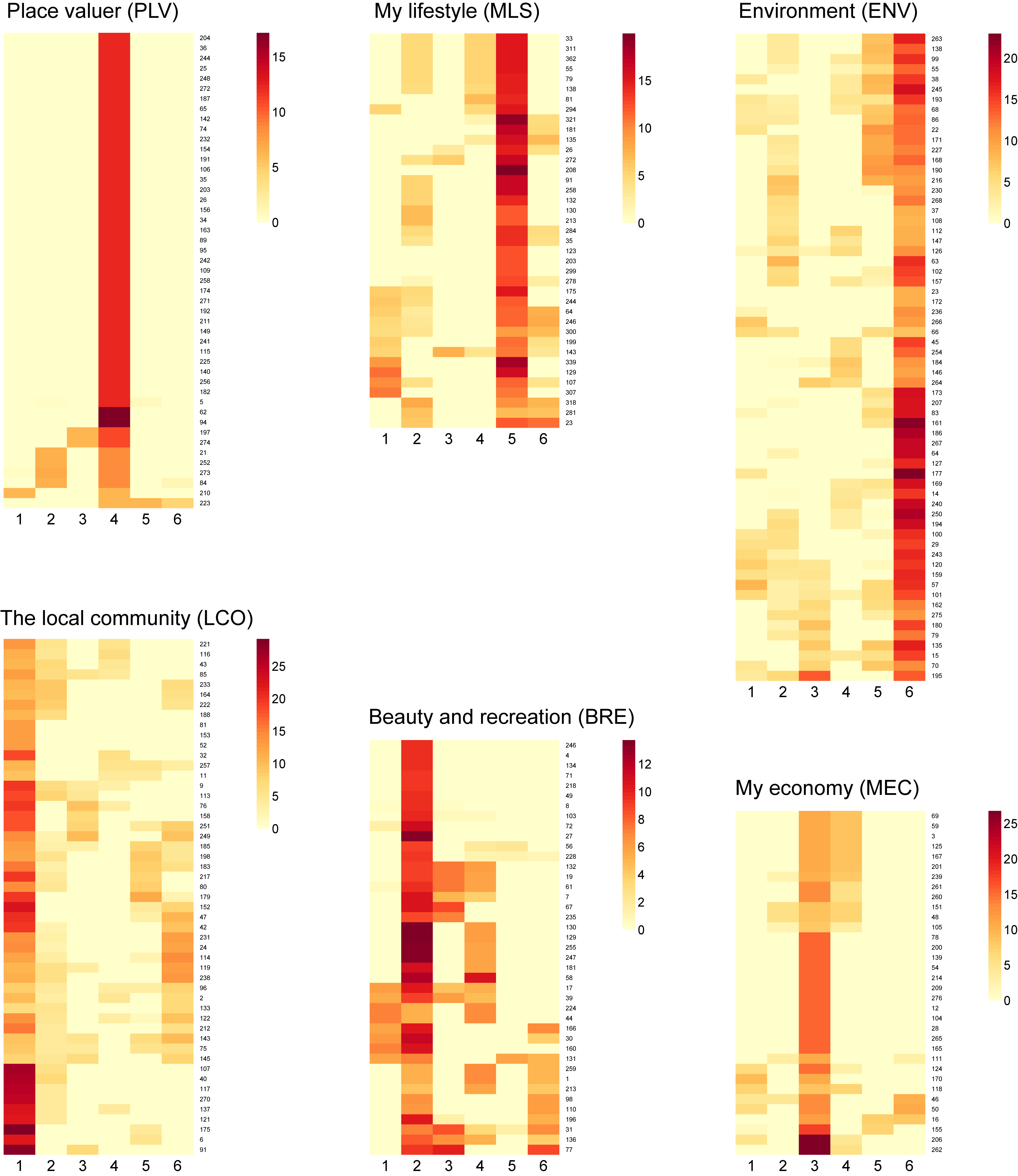

Supplement: S3 Fig — Groups are identified based on individual respondents' preference loadings (S1 Fig) on all value clusters identified (S2 Fig). Numbers 1 to 6 refer to value clusters 1 = "Focus on the local community", 2 = "Aesthetics", 3 = "Personal economy", 4 = "The place as such", 5 = "Active and conscious lifestyle choices", and 6 = "Nature and health" (S2 Table). Elaborate interpretations of the value profiles are provided in Table 2. (TIF) [file pone.0210426.s003.tif]

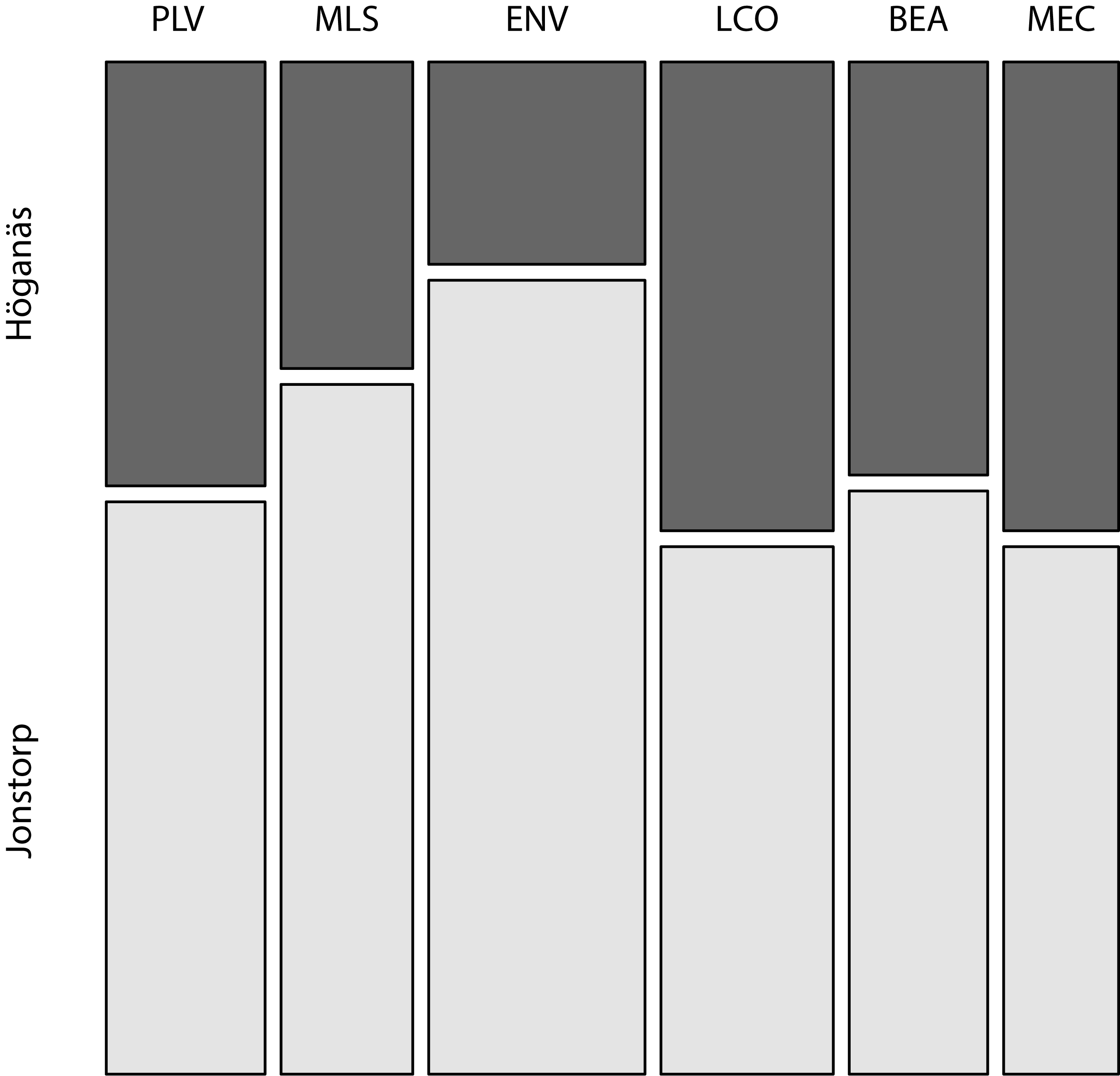

Supplement: S4 Fig — The size of the respective compartment is proportional to the number of observations in the respective category. PLV = "Place valuer", MLS = "My life style", ENV = "Environmental", LCO = "The local community", BEA = "Beauty", MEC = "My economy". The graph is based on raw data before imputation (χ = 12.96, n = 276, p = 0.023). (TIF) [file pone.0210426.s004.tif]

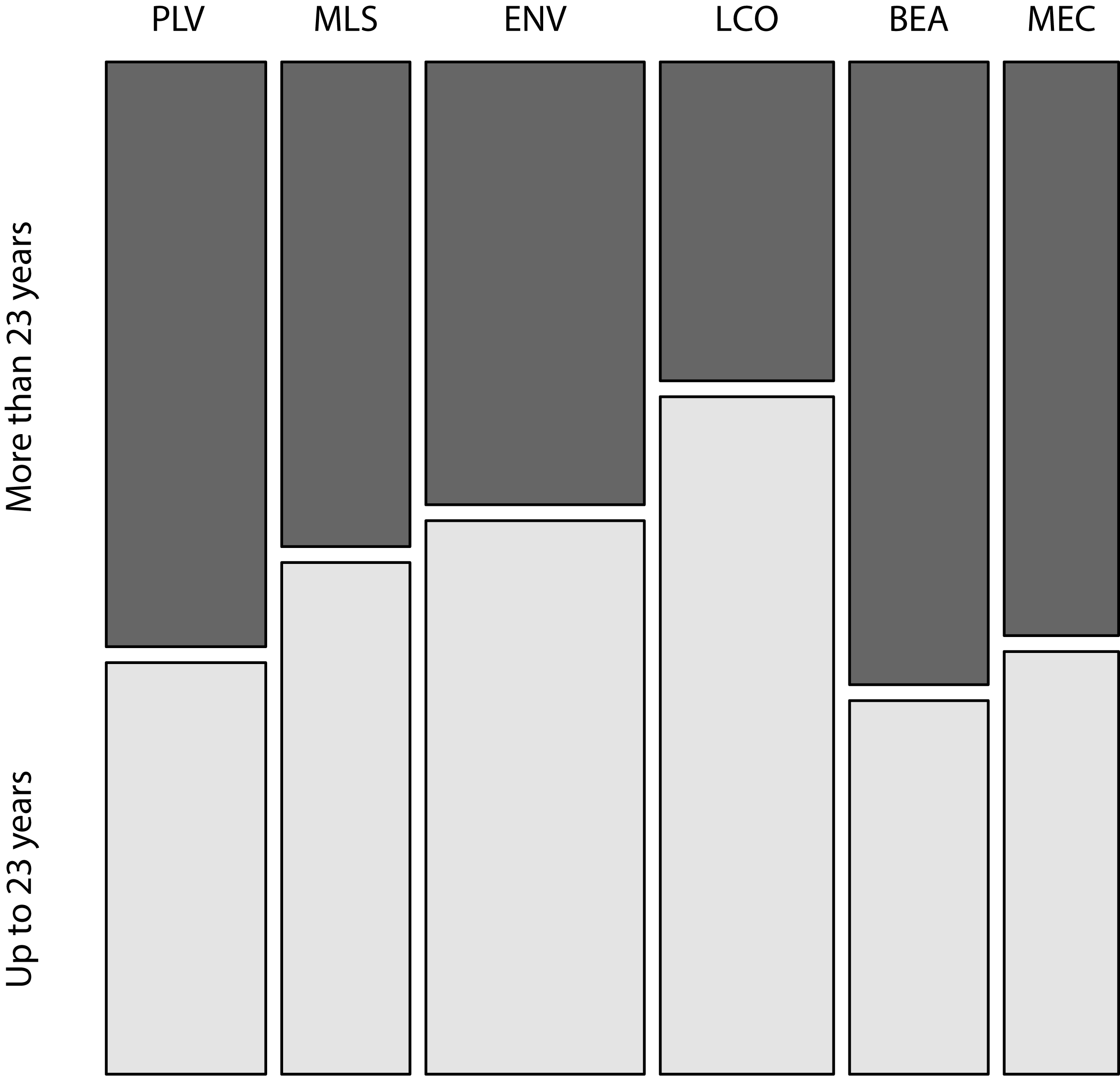

Supplement: S5 Fig — The size of the respective compartment is proportional to the number of observations in the respective category. PLV = "Place valuer", MLS = "My life style", ENV = "Environmental", LCO = "The local community", BEA = "Beauty", MEC = "My economy". The graph is based on raw data before imputation (χ2 = 11.90, n = 269, p = 0.035). (TIF) [file pone.0210426.s005.tif]

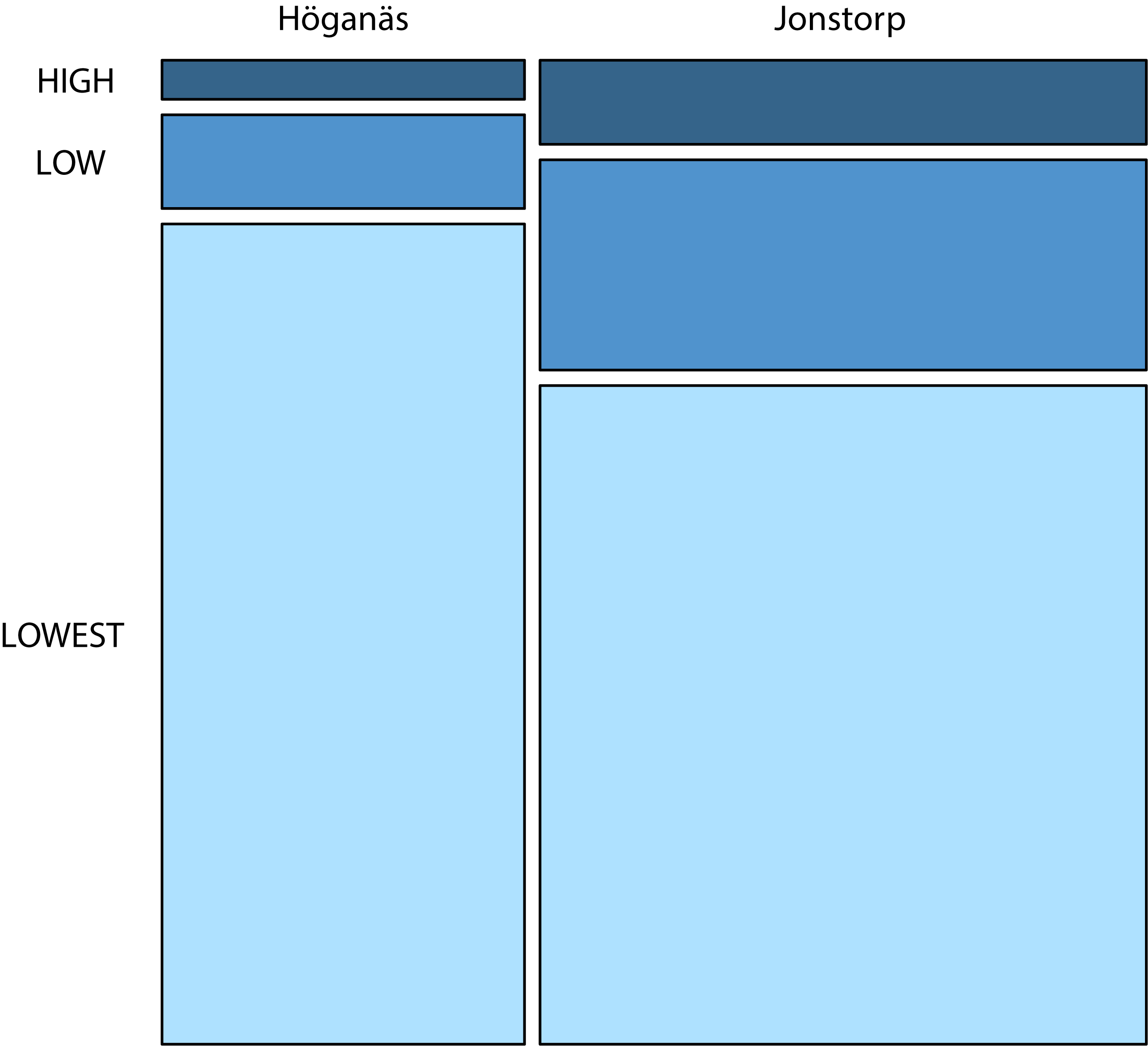

Supplement: S6 Fig — The graph is based on raw data before imputation (W = 10340, n = 324, p = 0.00067). (TIF) [file pone.0210426.s006.tif]

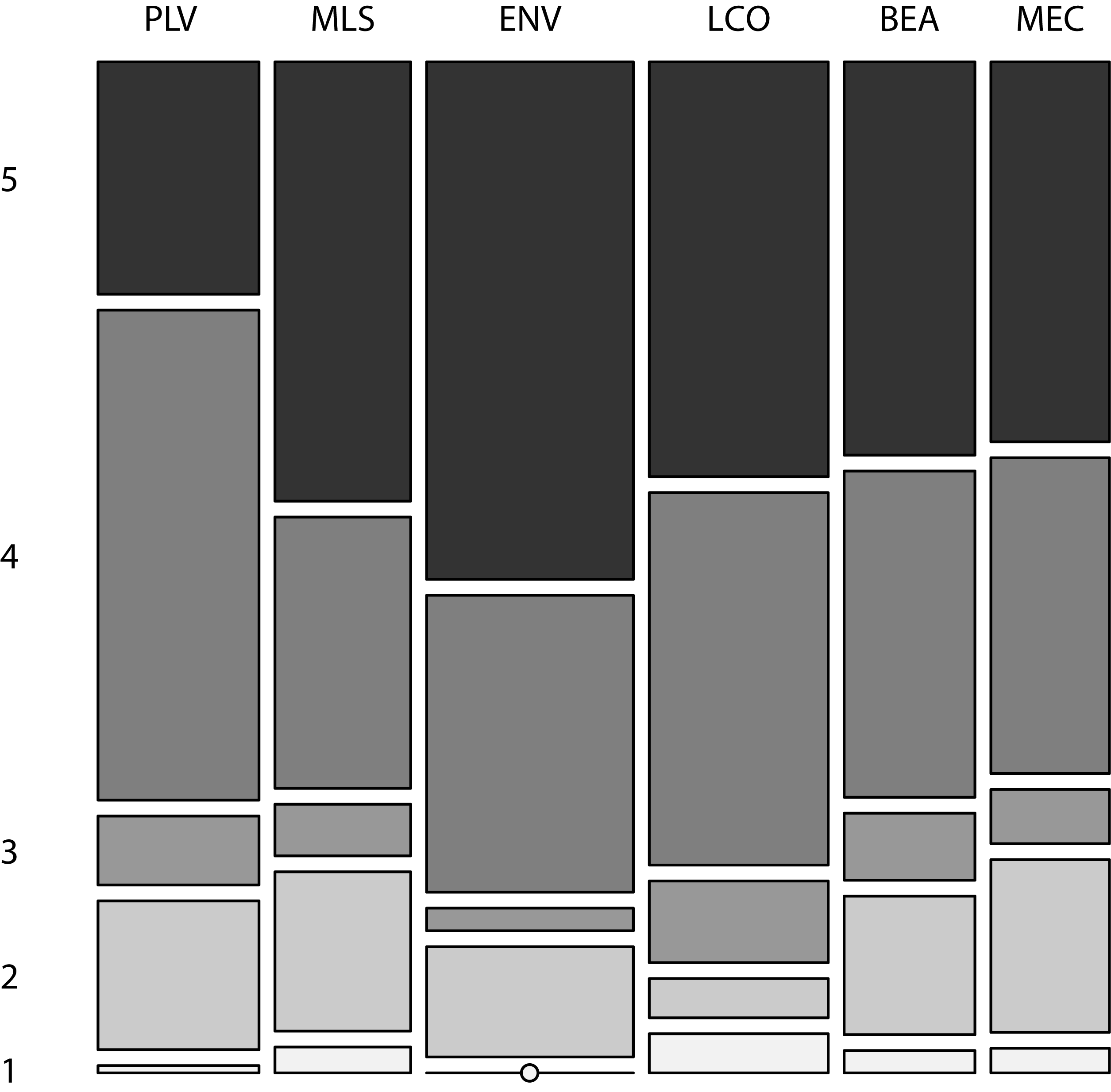

Supplement: S7 Fig — 1 refers to "Definitely not""; 2 "Probably not"; 3 "I do not know"; 4 "Yes, probably" and 5 "Yes, definitely". The size of the respective compartment is proportional to the number of observations in the respective category. PLV = "Place valuer", MLS = "My life style", ENV = "Environmental", LCO = "The local community", BEA = "Beauty", MEC = "My economy". (TIF) [file pone.0210426.s007.tif]

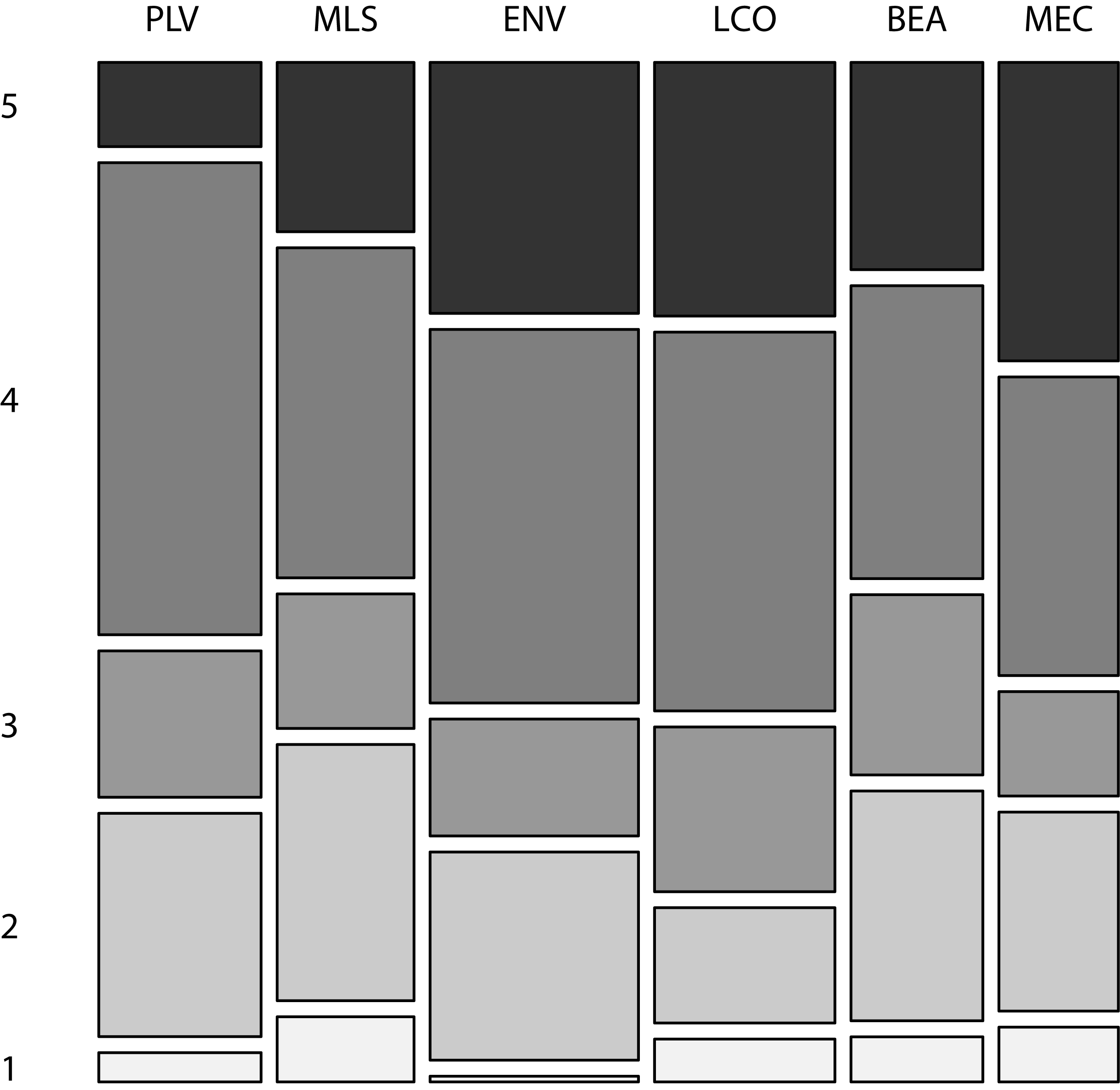

Supplement: S8 Fig — 1 refers to "Definitely not""; 2 "Probably not"; 3 "I do not know"; 4 "Yes, probably" and 5 "Yes, definitely". The size of the respective compartment is proportional to the number of observations in the respective category. PLV = "Place valuer", MLS = "My life style", ENV = "Environmental", LCO = "The local community", BEA = "Beauty", MEC = "My economy". (TIF) [file pone.0210426.s008.tif]

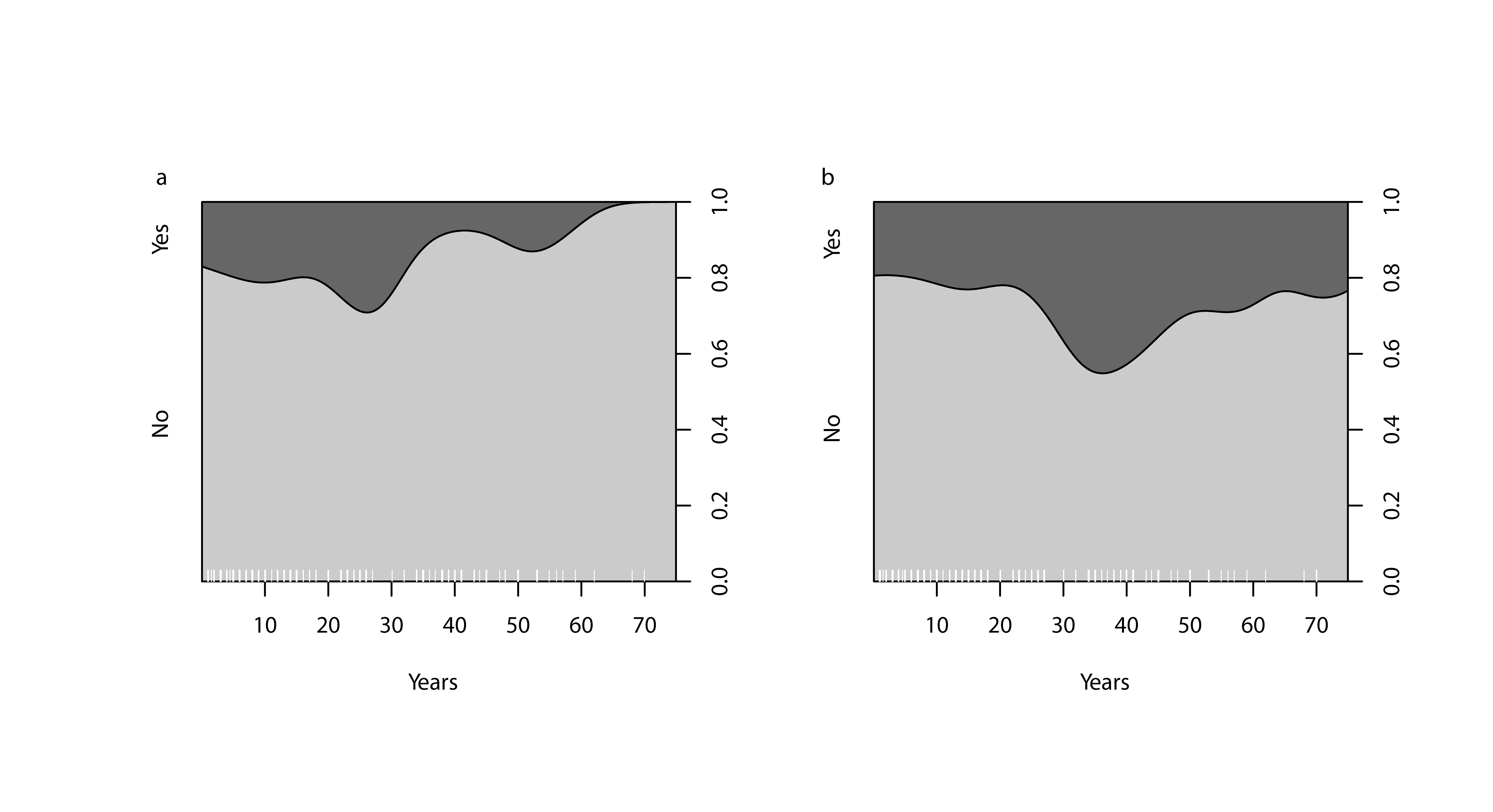

Supplement: S9 Fig — Relationship between for how long the respondent has lived in the municipality and reports of having experienced storm surge (a) and coastal erosion (b) because of climate change, respectively. Only responses from those respondents who answered "Yes, definitely" or "Yes, probably" to the question "Did you experience extreme weather or that the climate has changed in a way that you interpret as caused by long-term and global climate change?" were used (Table 1). The is no statistically significant difference between respondents having lived in the municipality for more than 23 years (median) or up to 23 years with respect to reporting to have experienced climate change induced storm surge (χ2 = 3.51, n = 179, p = 0.085). Respondents having lived in the municipality for more than 23 years (median) statistically significantly more often reported that they have experienced climate change induced coastal erosion than those having lived in the municipality for a period up to the median did (χ2 = 17.81, n = 177, p = 0.00002). Bars represent observations. Based on raw data before imputation. (TIF) [file pone.0210426.s009.tif]

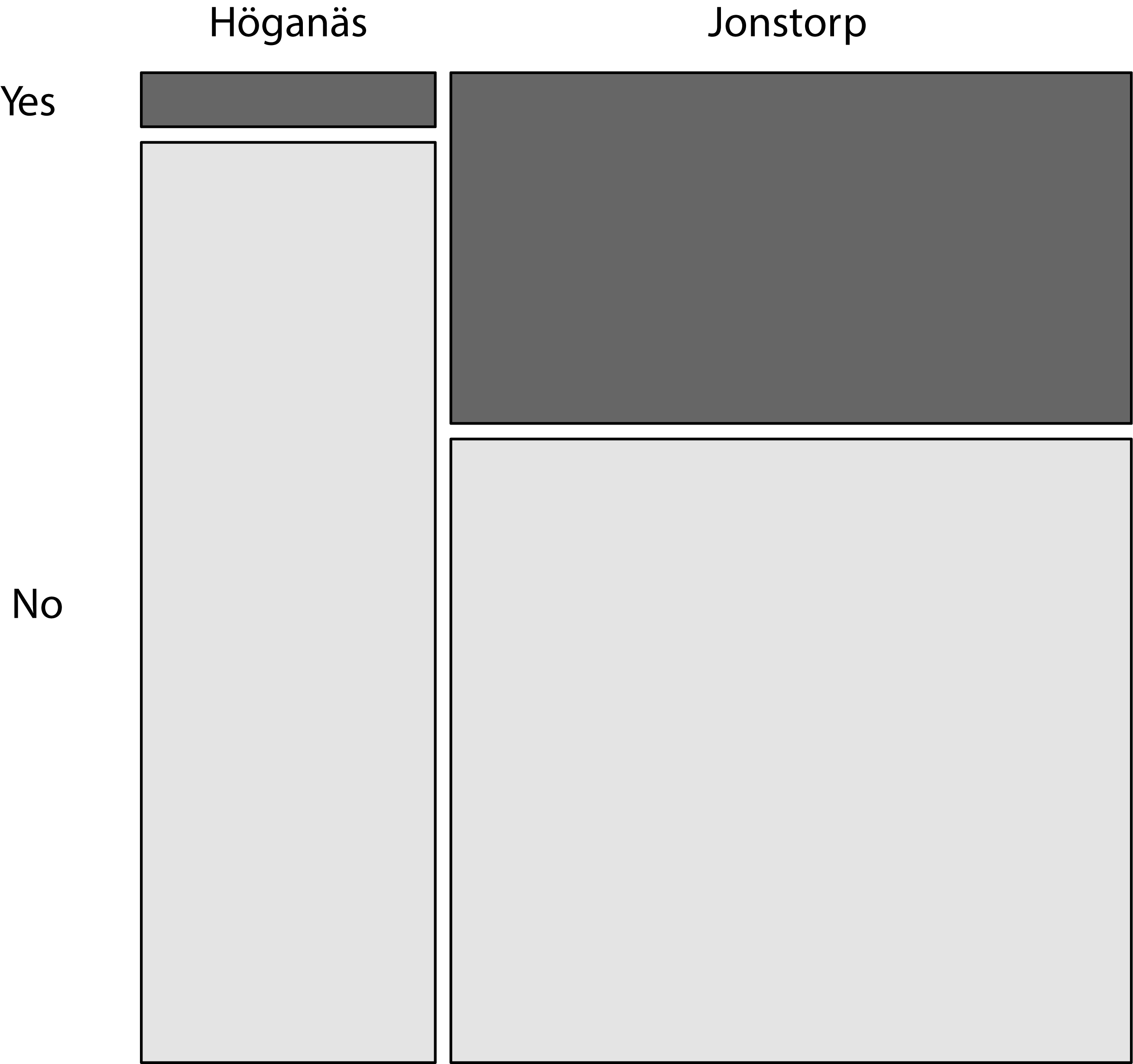

Supplement: S10 Fig — Only responses from those who answered "Yes, definitely" or "Yes, probably" to the question "Did you experience extreme weather or that the climate has changed in a way that you interpret as caused by longterm and global climate change?" (Table 1). Respondents living in Jonstorp statistically significantly more often reported that they have experienced climate change induced coastal erosion than those living in Höganäs did (χ2 = 17.81, n = 179, p = 0.00007). Based on raw data before imputation. (TIF) [file pone.0210426.s010.tif]
