# Supplementary material for: Are values related to culture, identity, community cohesion and sense of place the values most vulnerable to climate change?
Source: PLoS One. 2019 Jan 10;14(1):e0210426. doi: 10.1371/journal.pone.0210426 (PMC6328185; doi:10.1371/journal.pone.0210426)
Supplement: S1 Table — Classification is made in relation to the value categories 'culture', 'identity', 'community cohesion' and 'sense of place' in [3], the range observed (maximum 1–7) and median score of values assigned by n respondents (total n = 276). (PDF) [file pone.0210426.s011.pdf]

S1 Table. Predefined end values and classification. Classification is made in relation to the value categories 'culture', 'identity', 'community cohesion' and 'sense of place' in [3], the range observed (maximum 1-7) and median score of values assigned by n respondents (total n=276).

End value	***Value category***	***Range***	***Median/n***
Local economy	CC	1-7	6/68
Makes the place come alive	CC	1-7	6/65
The family	I	1-7	7/50
Bathing	O	1-7	6/34
Nice beach	O	1-7	6/25
Convenience	O	1-7	7/24
Vacation locally	CC	1-7	7/15
Socialice	O	1-7	5/19
Protection of economic values	O	1-7	6.5/4
Being with friends	I	1-7	5/9
Open landscape	SP	1-7	6.5/2
Berry and mushroom eating	O	1-7	6/5
Cooling bath	O	1-7	6/1
Sport	O	1-5	4.5/2
Aesthetics	C	1-7	6.25/150
Pride in one's region	I	1-7	6/74
Personal economy	O	1-7	6/51
Local history	SP	1-7	5/32
Community identity	I	1-7	7/23
Local traditions	C	1-7	5.5/114
Community cohesion	CC	1-7	6.5/8
National economy	O	1-7	5.25/22
New experiences	O	1-7	5/8
Integration	CC	1-7	6/102
The place as such	SP	1-7	6/131
Personal identity	I	1-7	5/9
Family traditions	I	1-7	6/5
Relaxation	O	1-7	6/128
Active lifestyle	O	1-7	6/66
Personal health	O	1-7	7/113
Personal weight concerns	O	1-7	6/11
Berry and mushroom picking	O	1-7	6/6
Knowing your home region	SP	1-6	5/6
Nature	O	1-7	7/96
Well-being	O	1-7	6.5/79
Experience nature	O	1-7	7/56
Biodiversity	O	1-7	7/65
Environmental concerns	O	1-7	7/34
Healthy environment	O	1-7	7/31
Plant watching	O	1-7	6/30
Local climate	SP	1-7	6/18
Meetings with wild animals	O	1-7	7/13
The future of humanity	O	1-7	7/71
Historical values	C	1-7	7/11
The future of the community	CC	1-7	7/6

Gardening	SP	1-7	7/6
Protection against erosion	O	1-7	7/64
Pollination	O	1-7	7/5
Agriculture	O	1-7	7/5
Using the gifts of nature	O	1-7	5/7
Artistic inspiration	C	1-7	5.5/6
Protection against flooding	O	1-7	6/3
Team sports	CC	1-7	5/4
Spiritual values	C	1-7	6/2
Excitement	O	-	-/0
Production of paper, books, building materials etc.	O	-	-/0
Reduced use of fossil fuels	O	-	-/0

C=Culture, I=Identity, CC=Community cohesion, SP=Sense of place, O=Other values.
