# Supplementary material for: Are values related to culture, identity, community cohesion and sense of place the values most vulnerable to climate change?
Source: PLoS One. 2019 Jan 10;14(1):e0210426. doi: 10.1371/journal.pone.0210426 (PMC6328185; doi:10.1371/journal.pone.0210426)
Supplement: S2 Table — (PDF) [file pone.0210426.s012.pdf]

S2 Table. Value cluster interpretation.

Value cluster	*Interpretation*	*Characterisation in relation to "culture, identity, community cohesion and sense of place", and other values ("personal well-being" and "economic/material value")*
1. Focus on the local community	This cluster is dominated by two values: The economic well-being of the local community (0.12), and bringing life to the local community (0.111). The focus here is thus clearly on the local community. Even though it is partly about economic well-being.This should be contrasted with other economic values that focus on the economic well-being of oneself and the country. It thus seems fair to categorise this concern as being about the local community rather than about economy as such.	Community cohesion
2. Aesthetics	This cluster is strictly dominated by one value, namely aesthetics (0.515). It also has an addition of values having to do with relaxation (0.158), though this value is very weak in relation to aesthetics.	Culture
3. Personal economy	This cluster is dominated by one value: Personal economy (0.242).	Economic/Material value
4. The place as such	This cluster is strictly dominated by one value: The value of the place as such, no matter what it can be used for (0.572).	Sense of place
5. Active and conscious lifestyle choices	This cluster is dominated by three types of value, all being strongly connected with active and conscious lifestyle choices: Relaxation (0.208), an active lifestyle (0.189) and personal health (0.145). The inclusion of both relaxation and an active lifestyle might on the surface look contradictory but in practice it seems to make sense. Being physically active can be relaxing for the mind and a period of physical activity typically calls for a period of relaxation and recovery.	Identity/Personal well-being
6. Nature and health	This cluster is also dominated by three value types: Appreciation of nature in its own right (and not for what one can get from it in the form of material values) (0.191), well-being (connected to a healthy environment) (0.132), and personal health (0.106). We can therefore	Sense of place/Personal well-being

	say that this cluster is focused on a concern for the health of oneself as well as for nature, the latter, both as an end value and as a means for one's own health. The appreciation of nature in its own right can be subsumed under the rubric "a sense of place" since it has to do with the particular nature in the particular place pointed out by the respondent.	

Values refer to loadings of individual end values on the value cluster in S1 Fig.
