# Supplementary material for: Are values related to culture, identity, community cohesion and sense of place the values most vulnerable to climate change?
Source: PLoS One. 2019 Jan 10;14(1):e0210426. doi: 10.1371/journal.pone.0210426 (PMC6328185; doi:10.1371/journal.pone.0210426)
Supplement: S3 Table — The components are strength of belief in local effects of climate change (Question 1 in Table 1) and the strength of belief in having experienced the effects of climate change (Question 2 in Table 1). (PDF) [file pone.0210426.s013.pdf]

S3 Table. Statistically significant relationships among variables in the models of risk perception components. The components are strength of belief in local effects of climate change (Question 1 in Table 1) and the strength of belief in having experienced the effects of climate change (Question 2 in Table 1).

Question	***For how long time did you live in Höganäs municipality?***	***Value profile "Place valuer" (PLV)***	***Value profile "Environmental" (ENV)***	***What is your highest qualification? (re-classified to non-university/unversity)***	***What is your gender?***	***Where in the municipality do you live?***	***Do you live (permanently) in the municipality?***
Do you think that the climate is changing because of human induced climate change to the extent that it will affect your environment?	z=-2.370, p=0.018	W=5292, p=0.025	W=9235, p=0.024	W=13516, p=0.0012	W=13170, p=0.013	-	-
Did you experience extreme weather or that the climate has changed in a way that you interpret as caused by long-term and global climate change?	z=-2.207, p=0.028	-	-	W=12865, p= 0.023	-	W=13171, p= 0.00068	W=6742, p=0.0088
For how long time did you live in Höganäs municipality?		-	-	t=4.05, df=297.5, p=0.00010	t = 2.33, df = 297.3, p=0.020	-	-
Value profile "Place valuer" (PLV)			-	-	-	-	-
Value profile "Environmental" (ENV)				-	χ^2=5.87, p=0.024	-	-
What is your highest					-	-	χ^2=18.35,

qualification? (re-classified to non-university/unversity)		p=0.000040
What is your gender?	-	-
Where in the municipality do you live?		χ^2=7.04, p=0.012
